# Metformin Attenuates Glucose‐Driven Hepatocellular Carcinoma Progression by Targeting GPD2‐Mediated Metabolic Plasticity

**DOI:** 10.1155/sci5/9372425

**Published:** 2026-10-27

**Authors:** Supakant Siripongsampan, Manusanun Kateyai, Kanadit Piriyapairoje, Jantanipa Hannam, Charupong Saengboonmee, Teeranai Ittiudomrak, Sukanya Jaroenporn, Sittiporn Pattaradilokrat, Chatchai Phoomak

**Affiliations:** ^1^ Department of Biology Faculty of Science Chulalongkorn University Bangkok 10330 Thailand chula.ac.th; ^2^ Bioinformatics and Computational Biology Program Graduate School Chulalongkorn University Bangkok 10330 Thailand chula.ac.th; ^3^ Department of Biochemistry Faculty of Medicine Khon Kaen University Khon Kaen 40002 Thailand kku.ac.th; ^4^ Center of Excellence in Systems Biology Faculty of Medicine Chulalongkorn University Bangkok 10330 Thailand chula.ac.th

**Keywords:** cancer aggressiveness, drug repurposing, glycerol-3-phosphate dehydrogenase 2, liver cancer, metformin

## Abstract

Glucose metabolism is crucial for cancer cell survival and proliferation. Hepatocellular carcinoma (HCC) cells exhibit distinct metabolic adaptations that support tumor progression under nutrient‐limited conditions. The study examines the impact of glucose availability and GPD2‐mediated metabolic adaptation on HCC progression, as well as how these processes are modulated by glucose transport inhibitors and metformin. Cell proliferation and migration of HCC cells (HepG2 and Huh7) and lung cancer cells (A549) were examined under different glucose concentrations using MTT, colony formation, and scratch assays. The impacts of glucose transporter 1 (GLUT1) inhibitors and metformin were also evaluated. Gene and protein expression data were analyzed from TCGA/CPTAC datasets, and GPD2 expression was validated by immunohistochemistry. High glucose enhanced proliferation and migration of liver cancer cells. Unlike A549, HepG2 and Huh7 cells maintained growth under glucose deprivation, indicating metabolic plasticity. GPD2 was markedly upregulated in HCC tissues and correlated with poor survival. Metformin suppressed cell proliferation and migration in a dose‐dependent manner, with stronger effects under low‐glucose conditions. Mechanistically, metformin downregulated Cyclin D1 and XIAP, induced Caspase‐3 cleavage, and reduced the EMT marker, Slug. Correlation analyses revealed positive associations between GPD2 and CCND1 or SNAI2, indicating a link between GPD2 and cell cycle and EMT signaling. In conclusion, HCC cells utilize GPD2‐mediated metabolic adaptation to sustain growth under glucose‐limiting conditions. GPD2 represents a prognostic biomarker and therapeutic target in HCC. Metformin effectively inhibits HCC progression by disrupting GPD2‐linked pro‐survival and EMT pathways.

## 1. Introduction

Hepatocellular carcinoma (HCC) is the most common primary liver malignancy and a leading cause of cancer‐related mortality worldwide [1]. Despite advances in surgical resection, locoregional therapies, and systemic treatments, the prognosis of HCC patients remains poor due to late diagnosis, high recurrence rates, and limited therapeutic options for advanced disease [1]. A defining hallmark of HCC progression is metabolic reprogramming, an adaptive process that enables tumor cells to survive and proliferate in fluctuating nutrient environments characteristic of the liver tumor microenvironment [2–4].

Glucose metabolism plays a central role in cancer cell growth and survival. Many cancers exhibit enhanced glucose uptake and glycolysis, even in the presence of oxygen, a phenomenon known as the Warburg effect [5]. However, liver cancer cells possess additional metabolic flexibility because hepatocytes are intrinsically equipped with pathways such as gluconeogenesis, glycogen metabolism, and lipid turnover [6, 7]. This metabolic plasticity likely allows HCC cells to sustain proliferation under glucose‐limiting conditions; however, the specific molecular determinants that govern this adaptive metabolic shift remain poorly defined.

The glycerol‐3‐phosphate (G3P) shuttle is a critical metabolic pathway that links glycolysis, gluconeogenesis, and lipid metabolism while maintaining cellular redox balance [8–10]. A key component of this shuttle, mitochondrial glycerol‐3‐phosphate dehydrogenase (GPD2), catalyzes the oxidation of G3P to dihydroxyacetone phosphate (DHAP), thereby coupling cytosolic NADH reoxidation to mitochondrial oxidative phosphorylation [8–10]. In hepatic physiology, GPD2 is essential for glycerol‐derived gluconeogenesis and energy homeostasis [11, 12]. Although emerging evidence suggests that dysregulation of GPD2 may promote tumorigenesis by supporting metabolic rewiring, its specific role in HCC progression and patient prognosis has not been systematically explored.

Metformin, a widely prescribed antidiabetic drug, has gained increasing attention for its anticancer potential [13]. Epidemiological studies indicate that metformin use is associated with reduced cancer incidence and improved outcomes in several malignancies, including liver cancer [13]. Mechanistically, metformin inhibits mitochondrial complex I, disrupts oxidative metabolism, and induces energetic stress in cancer cells. Intriguingly, emerging evidence suggests that metformin‐mediated GPD2 inhibition disrupts mitochondrial redox shuttling, potentially independent of its effects on Complex I [14–16]. However, how metformin interacts with glucose availability and GPD2‐mediated metabolic adaptation in HCC remains unclear.

In this study, we investigated the role of glucose availability in liver cancer progression and identified GPD2 as a central mediator of metabolic plasticity in HCC. Integrating in vitro functional assays with multiomic clinical datasets and immunohistochemical analyses, we demonstrate that GPD2 is markedly upregulated in HCC and correlates with poor clinical outcomes. Furthermore, we show that metformin suppresses glucose‐driven ATP production, HCC proliferation, migration, and EMT signaling, with enhanced efficacy under low‐glucose conditions. Our findings establish GPD2 as a prognostic biomarker and therapeutic vulnerability in hepatocellular carcinoma and provide mechanistic insight into the glucose‐dependent anticancer effects of metformin.

## 2. Materials and Methods

### 2.1. Cell Culture and Treatment

HCC cell lines (Huh7 and HepG2) and lung adenocarcinoma cell line (A549) were cultured in DMEM medium supplemented with 10% FBS and antibiotics at 37°C in 5% CO_2_. The cells were tested for mycoplasma contamination every 6 months. To examine the effect of glucose concentration on liver cancer aggressiveness, liver cancer cells were cultured in media containing different glucose concentrations. The cells were then subjected to assays comparing cell growth and migration between conditions. To test the effect of metformin on liver cancer aggressiveness, the cells were treated with various concentrations of metformin before assessing cell growth and migration. To investigate the synergistic effect of metformin with glucose reduction, liver cancer cells were cultured under low and high glucose conditions, followed by treatment with metformin at high, medium (IC_50_), and low concentrations. Cell growth and the potential synergistic effect between glucose reduction and metformin treatment were then analyzed.

### 2.2. Suppression of Slug Expression Using siRNA

The expression of Slug in HCC cells was suppressed using siRNA specific to Slug (*SNAI2*; 5′‐UUGACCUGUCUGCAAAUGCTT‐3′). The cells (3 × 10^5^ cells/well) were cultured in a 6‐well plate for 24 h and then transfected with 100 pmol of si‐Slug using 2 μg/mL Lipofectamine 2000 (Invitrogen) according to the recommendations from the manufacturer. Excess transfection complex was removed after 6 h, and cells continued to be cultured in 10% FBS in DMEM for the subsequent experiments. Control experiments were performed using cells treated with negative control siRNA (negative control siRNA, 1027310, Qiagen).

### 2.3. Cell Proliferation Assay

To determine the proliferation rate of HCC cells after treatment, 3 × 10^3^ cells/well were seeded into a 96‐well plate and incubated overnight. After treating the cells with indicated compounds, cell numbers were determined at 0, 1, 3, and 5 days using 3‐(4,5‐dimethylthiazol‐2‐yl)‐2,5‐diphenyltetrazolium bromide (MTT) proliferation assay (Invitrogen, MA) according to the manufacturer’s recommendation. Briefly, MTT reagent was added to each well to reach a final concentration of 0.5 mg/mL. After 4 h incubation, the insoluble formazan complex was dissolved by 100 μL DMSO, and the absorbance at 570 nm was measured. Relative cell proliferation (fold change to day 0) was calculated.

### 2.4. Clonogenic Assay

To determine the single‐cell survival of HCC cells, 400 cells/well were seeded into a 6‐well plate. After overnight incubation, the cells were treated with indicated conditions for 14 days. Colonies were then fixed and stained with 0.5% crystal violet. The stained colonies of at least 50 cells were counted.

### 2.5. Cell Migration Assay

The migration assay was performed using Transwell (8.0 μm pore size, Corning Incorporated, Corning, NY) as described previously. After 24 h of treatment, 4 × 10^4^ HCC cells in 200 μL of serum‐free DMEM were added into each insert. Cells were allowed to migrate through the membrane to the lower chamber for 24 h. The migrated cells were stained with 0.5% crystal violet. Cells were counted under the microscope using a 10X objective lens with 5 microscopic fields/sample.

### 2.6. Wound Healing Assay

Cell migration was assessed using a wound healing assay. Briefly, 3.5 × 10^5^ cells were seeded into 24‐well plates and cultured until a confluent monolayer was formed. A single scratch was made in each well using a sterile pipette tip, and detached cells were removed by washing with PBS. The remaining cells were then incubated at 37°C to allow migration. Images of the wound area were captured at 0, 6, 12, and 24 h using an inverted microscope. The migration distance was quantified using ImageJ software.

### 2.7. Intracellular ATP Detection

The cells were treated with metformin or GPD2 inhibitor (KM04416) for 24 h. After treatment, intracellular ATP levels were measured using an ATP Assay Kit (HY‐K0314, MedChemExpress, NJ, USA), following the manufacturer’s protocols. Briefly, the cells were gently washed twice with cooled PBS and lysed with ATP lysis buffer. The lysates were then centrifuged at 12,000 × g for 5 min at 4°C, and the supernatants were collected. The ATP content of the samples was measured, and the luminescence signals were immediately detected using a Feyond‐A300 microplate reader (Allsheng, China).

### 2.8. SDS‐Polyacrylamide Gel Electrophoresis (PAGE) and Western Blot Analysis

SDS‐PAGE and Western blot were performed to determine the levels of protein expression. Cells were harvested in RIPA lysis buffer (Thermo Scientific, Waltham, MA) containing phosphatase and protease inhibitors. Protein concentration was determined using Pierce BCA Protein Assay (Thermo Scientific). The samples for SDS‐PAGE were prepared by mixing cell lysates with a sample buffer containing 2‐mercaptoethanol. Protein (10–20 μg) was loaded into a 10% SDS‐polyacrylamide slab gel. After electrophoresis, the separated proteins were then transferred onto a PVDF membrane.

### 2.9. Immunodetection

After electro‐transferred, the PVDF membranes were washed twice with washing buffer (0.3% Tween‐20 in PBS pH 7.4, PBST) and nonspecific binding was blocked by incubating with 5% skim milk in PBST for 1 h at room temperature. After blocking, the membranes were incubated overnight at 4°C with the indicated primary antibodies (Table S1). After three washes, the membranes were incubated with the corresponding secondary antibody. The immunoreactivity was detected by incubating with Clarity Western ECL Substrate (Bio‐Rad Laboratories Ltd, Hercules, CA). The chemiluminescent signal was captured by ChemiDoc analyzer (Bio‐Rad), and the signal intensities were quantified by ImageJ software. GAPDH was concomitantly detected and used as an internal control.

### 2.10. Gene Expression Profile and Meta‐Analysis

The expression profiles of GPD1, GPD2, and their correlation with clinical data of the patients were retrieved from publicly available databases: the University of Alabama at Birmingham Cancer (UALCAN) data analysis portal (https://ualcan.path.uab.edu/) [17] and the Gene Expression Profiling Interactive Analysis (GEPIA; https://gepia.cancer-pku.cn/index.html) [18]. A dataset on GPD1 and GPD2 protein expression in tumors compared to normal tissues was extracted via the UALCAN database, using the integrative data from the Clinical Proteomic Tumor Analysis Consortium (CPTAC) and the International Cancer Proteogenome Consortium (ICPC). The correlations between the genes in HCC were explored using GEPIA. Immunohistochemistry (IHC) scores of GPD1 and GPD2 in normal and liver cancer tissues were explored via the Human Protein Atlas (HPA) database (https://www.proteinatlas.org/) [19].

### 2.11. Statistical Analysis

Statistical analysis was determined using Student’s *t*‐test for the pair‐tested/unpaired‐tested and one‐way or two‐way ANOVA followed by Tukey’s multiple comparisons test for the group analysis (GraphPad Prism v9.5 software; GraphPad software Inc., La Jolla, CA). For survival analysis, patients were stratified into high‐ and low‐GPD2 expression groups using the median GPD2 expression level as the cut‐off. Overall survival was then evaluated using the Kaplan–Meier method, and differences between groups were compared using the log‐rank test. To identify independent prognostic factors for overall survival, the proportional hazards (PH) assumption was assessed for each categorical variable using log‐minus‐log plots. The PH assumption was considered satisfied when the curves for the respective groups were approximately parallel over time. Univariate Cox proportional hazards regression analyses were then performed for each variable, followed by multivariate Cox proportional hazards regression analysis to identify independent prognostic factors. The survival analyses were performed using IBM SPSS Statistics Version 30.0 (IBM Corp., Armonk, NY, USA). A two‐sided *p* < 0.05 was considered statistically significant.

## 3. Results

### 3.1. High Glucose Enhances Proliferation and Migration of Cancer Cells

To investigate whether the metabolic adaptation to glucose deprivation is a unique characteristic of hepatocellular carcinoma (HCC), we compared two HCC cell lines (Huh7 and HepG2) with the lung adenocarcinoma cell line A549, which is known to exhibit a relatively high dependence on glucose metabolism. The cells were cultured under different glucose conditions (0, 1, 5, and 10 mM) and the cell proliferation was determined using MTT assay. A549, Huh7, and HepG2 cells showed significantly higher proliferation under high glucose (10 mM) conditions compared to low or no glucose conditions, indicating that glucose availability strongly promotes cancer cell growth (Figure 1A). However, the inhibitory effect of low glucose on cancer cell proliferation was predominantly observed in A549 cells.

**FIGURE 1:**
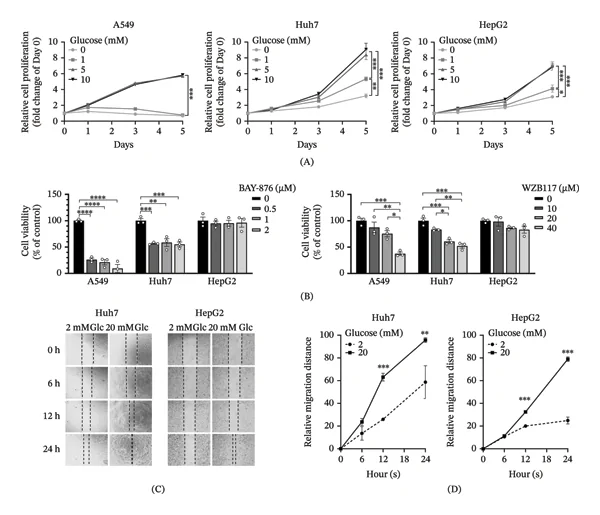
High glucose promotes the progression of hepatocellular carcinoma and lung cancer cells. (A) A549, Huh7, and HepG2 cells were cultured in media containing 0, 1, 5, or 10 mM glucose, and cell proliferation was measured by MTT assay on days 0, 1, 3, and 5. (B) The viability of A549, Huh7, and HepG2 cells treated with the indicated concentrations of GLUT1 inhibitors, BAY‐876 or WZB117, was assessed by MTT assay. (C) Representative images of scratch‐wound assays showing the effect of glucose on the migration of Huh7 and HepG2 cells. (D) Quantification of wound closure in Huh7 and HepG2 cells cultured in 2 or 20 mM glucose. Data are presented as mean ± SEM from three independent experiments. Statistical significance was determined by one‐way ANOVA followed by Tukey’s multiple‐comparisons test for (A) and (B) and by Student’s *t*‐test for (D) (^∗^ *p* < 0.05, ^∗∗^ *p* < 0.01, ^∗∗∗^ *p* < 0.001, ^∗∗∗∗^ *p* < 0.0001).

To investigate the effect of intracellular glucose on the survival of liver and lung cancer cells, the cells were treated with selective glucose transporter 1 (GLUT1) inhibitors—BAY‐876 and WZB117—and cell survival was assessed using MTT assay. As shown in Figure 1B, BAY‐876 treatment markedly reduced the viability of A549 cells but had minimal effects on Huh7 and HepG2 cells, suggesting a cell type‐specific dependence on GLUT1. Similarly, WZB117 treatment reduced A549 cell viability in a dose‐dependent manner but produced less pronounced effects in Huh7 and HepG2 cells, suggesting that these liver cancer cells may utilize alternative glucose uptake pathways or undergo metabolic adaptation.

The migratory ability of liver cancer cells cultured under 2 and 20 mM glucose conditions was further evaluated using a wound healing assay. As shown in Figure 1C, high glucose significantly enhanced the migratory capacity of HCC cells compared with low‐glucose conditions. Together, these findings suggest that elevated glucose promotes aggressive phenotypes in HCC cells.

### 3.2. GPD2 is Upregulated in HCC

Although glucose availability significantly influenced HCC cell proliferation, the cells retained the ability to proliferate even under glucose‐deprived conditions. These findings led us to hypothesize that HCC cells may access alternative glucose sources or activate compensatory metabolic pathways, such as gluconeogenesis, to sustain intracellular glucose levels. As illustrated in Figure 2A, glycogen metabolism, gluconeogenesis, and lipid metabolism are closely interconnected and may together contribute to the maintenance of intracellular glucose homeostasis.

FIGURE 2GPD2 is upregulated and associated with poor prognosis in hepatocellular carcinoma. (A) Schematic illustration of the metabolic interplay among glycogen metabolism, glycerol‐derived gluconeogenesis, and lipid metabolism, highlighting the roles of GPD1 and GPD2 in glycerol‐3‐phosphate (G3P) metabolism and the proposed inhibitory effect of metformin on GPD2. (B) mRNA expression (TCGA) and protein expression (CPTAC) of GPD1 and GPD2 in normal liver and hepatocellular carcinoma (HCC) tissues. (C) mRNA and protein expression of glycogen metabolism‐related genes (PYGL, PYGB, and GYS2) in normal liver and HCC tissues. (D) Pearson correlation analysis between GPD1 and GPD2 mRNA expression in HCC tissues. (E) Representative immunohistochemical (IHC) staining images of GPD1 and GPD2 in normal liver and HCC tissues obtained from the Human Protein Atlas (HPA), with enlarged views of the boxed regions shown below. (F) Quantitative comparison of GPD1 and GPD2 IHC staining scores in normal liver and HCC tissues; ^∗^ *p* < 0.05, ^∗∗^ *p* < 0.01, and two‐way ANOVA followed by Tukey’s multiple comparisons test. (G) Paired analysis of GPD1 and GPD2 IHC scores within the same HCC specimens. Kaplan–Meier survival analyses showing the association of GPD1 (H) and GPD2 (I) expression with overall survival (OS) and disease‐free survival (DFS) in patients with HCC. Statistical significance is indicated in each panel as provided by the respective databases.

**Figure:**
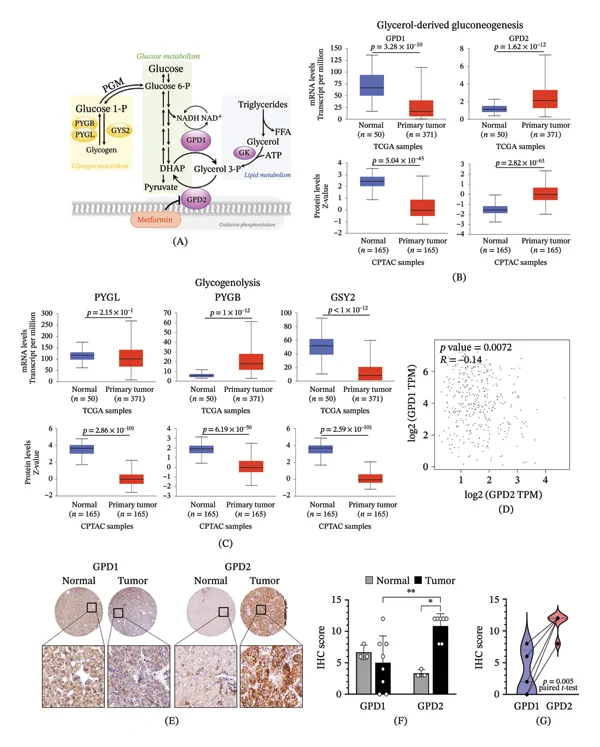

**Figure:**
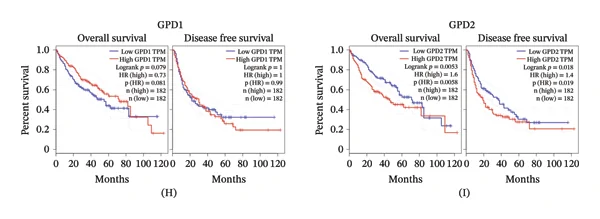

The expression levels of genes involved in glycerol‐derived gluconeogenesis and glycogen metabolism were retrieved and explored via GEPIA. Analysis of glycerol‐derived gluconeogenesis revealed differential expression of GPD1 and GPD2 between tumor and normal tissues across cancer types (Figure S1A). GPD1 was generally reduced in tumors, whereas GPD2 showed broader expression and was frequently elevated, indicating a shift in glycerol metabolic utilization in cancer. Genes involved in glycogen metabolism were also markedly dysregulated (Figure S1B). GYS2 expression was consistently lower in tumors compared with normal tissues, while glycogenolytic enzymes PYGB and PYGL were broadly expressed and frequently upregulated in tumors, with PYGL showing pronounced variability and elevation in multiple cancers.

The differential expression of these genes between HCC tumor and normal liver tissues was further analyzed using publicly available cohort databases. As shown in Figure 2B, analysis of TCGA and CPTAC datasets revealed that both GPD2 mRNA and protein levels were significantly upregulated in HCC tumor tissues compared with normal liver tissues, whereas GPD1 expression was markedly downregulated. Protein expression of PYGB, PYGL, and GSY2, was significantly reduced in tumor samples, indicating the reduction of glycogen‐glucose recycling in HCC (Figure 2C). These findings indicate translational activation of gluconeogenesis‐related enzymes in HCC, supporting metabolic adaptation associated with tumor growth.

The relationship between GPD1 and GPD2 expression was further examined using Pearson correlation analysis. As shown in Figure 2D, GPD1 expression was negatively correlated with GPD2 expression in liver cancer tissues (*R* = −0.14, *p* = 0.007). To further validate the transcriptomic and proteomic findings obtained from the TCGA and CPTAC datasets, immunohistochemical (IHC) data from The HPA were analyzed. As shown in Figure 2E, both enzymes were detectable in normal liver and tumor tissues; however, GPD2 exhibited markedly stronger staining intensity in tumor sections, whereas GPD1 showed only modest changes. Quantitative analysis of IHC scores confirmed that GPD2 expression was significantly elevated in HCC compared with normal tissues, while GPD1 displayed no changes (Figure 2F). Moreover, paired analysis demonstrated consistently higher GPD2 expression relative to GPD1 within the same tumor specimens (Figure 2G). Collectively, these results indicate preferential overexpression of GPD2 in HCC, suggesting a more prominent role for GPD2 than GPD1 in liver cancer progression.

### 3.3. High GPD2 Expression Is Associated With Poor Clinical Outcome of the HCC Patients

The relationship between the expression of genes involved in glycerol‐derived gluconeogenesis and glycerol metabolism and patient survival was evaluated. Survival analyses revealed significant correlations between metabolic gene expression and clinical outcomes (Figure S1C). High expression of GPD2, PYGB, and PYGL was generally associated with unfavorable overall and disease‐free survival, whereas elevated GPD1 and GYS2 expression correlated with improved clinical outcomes in a cancer type‐dependent manner.

Kaplan–Meier survival analysis revealed that patients with high GPD2 expression had significantly poorer overall and disease‐free survival than those with low GPD2 expression (*p* = 0.005 and *p* = 0.018, respectively), whereas GPD1 expression showed a trend toward reduced survival but did not reach statistical significance (Figure 2H,I). To determine whether GPD2 expression is an independent prognostic factor, univariate and multivariate Cox proportional hazards regression analyses were performed using TCGA clinical data. Univariate analysis identified advanced T, N, and M stages, as well as high GPD2 expression, as significant predictors of poor overall survival (Table 1). Importantly, after adjustment for age, sex, T stage, N stage, and M stage, high GPD2 expression remained independently associated with unfavorable overall survival (HR = 1.619, 95% CI = 1.110–2.361, *p* = 0.012), together with advanced T stage (HR = 3.189, 95% CI = 2.198–4.628, *p* < 0.001) (Table 1). These results demonstrate a significant association between GPD2 expression and patient survival, suggesting that GPD2 may serve as both an independent prognostic indicator and a potential therapeutic target in HCC.

**TABLE 1:** Univariate and multivariate analysis of factors influencing overall survival in HCC patients.

Variables (*N*)	Univariate analysis			Multivariate analysis		
	HR	95% CI	*p* value	HR	95% CI	*p* value
Age						
≤ 60 (165)	1			1		
> 60 (167)	1.095	0.758–1.580	0.629	1.027	0.704–1.497	0.891
Sex						
Male (228)	1			1		
Female (104)	0.854	0.585–1.247	0.415	0.900	0.615–1.316	0.587
T stage						
T1‐T2 (245)	1			1		
T3‐T4 (87)	3.030	2.097–4.378	**< 0.001**	3.189	2.198–4.628	**< 0.001**
N stage						
N0 (236)	1			1		
NX (96)	1.498	1.015–2.211	**0.042**	1.426	0.859–2.368	0.170
M stage						
M0 (241)	1			1		
MX (91)	1.605	1.084–2.376	**0.018**	1.366	0.825–2.261	0.226
GPD2						
Low (164)	1			1		
High (168)	1.617	1.116–2.343	**0.011**	1.619	1.110–2.361	**0.012**

*Note:* Bold values indicate statistical significance in the table.

### 3.4. Differential Expression of Glycerol Metabolism and Glycogenolysis Genes in Lung Cancer and HCC Cells

The expression of genes involved in glycerol‐derived gluconeogenesis and glycogen metabolism was further examined in lung cancer (A549) and HCC cell lines (Huh7 and HepG2). Transcriptomic analysis revealed distinct regulation of glycerol‐derived gluconeogenesis genes between the three cell lines (Figure 3A). GPD1 expression was markedly higher in HepG2 cells, whereas GPD2 expression predominated in A549 and Huh7 cells. In contrast, PYGL and PYGB were expressed at comparable levels in all cell lines, while GYS2 expression was undetectable (Figure 3B). Differential expression of GPD1 and GPD2 was confirmed at the protein level by Western blot analysis (Figure 3C). GPD1 protein was abundantly expressed in HepG2 cells but was minimally detected in A549 and Huh7 cells. In contrast, GPD2 protein expression was markedly higher in A549 and Huh7 cells than in HepG2 cells.

**FIGURE 3:**
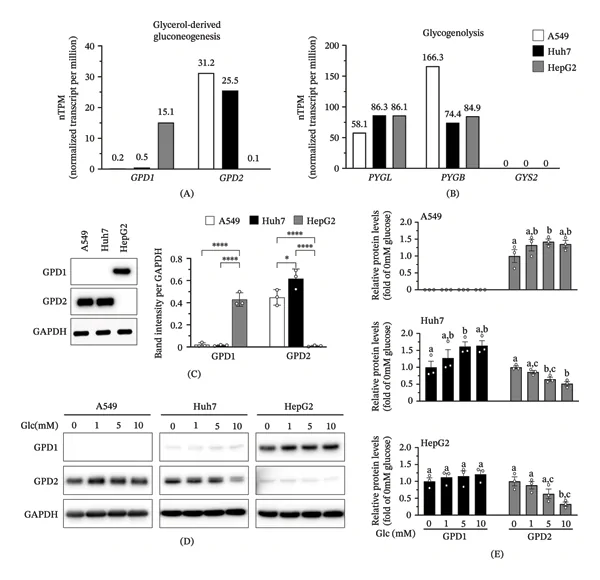
Differential expression of glycerol metabolism‐related genes in A549, Huh7, and HepG2 cells and their regulation by glucose availability. Basal mRNA expression levels of (A) glycerol‐derived gluconeogenesis‐related genes (GPD1 and GPD2) and (B) metabolism‐related genes (PYGL, PYGB, and GYS2) in A549, Huh7, and HepG2 cells, determined from transcriptomic data. (C) Representative Western blot analysis of GPD1 and GPD2 protein expression in A549, Huh7, and HepG2 cells, with GAPDH used as the loading control. Bar graph presents densitometric quantification of GPD1 and GPD2 protein expression normalized to GAPDH. Data are presented as mean ± SEM; one‐way ANOVA followed by Tukey’s multiple comparisons test (^∗^ *p* < 0.05, ^∗∗∗∗^ *p* < 0.0001). (D) Representative Western blot analysis of GPD1 and GPD2 protein expression in A549, Huh7, and HepG2 cells cultured under increasing glucose concentrations (0, 1, 5, and 10 mM). (E) Densitometric quantification of GPD1 and GPD2 protein expression, normalized to GAPDH and expressed relative to cells cultured in 0 mM glucose. Data are presented as the mean ± SEM. Bars labeled with different letters indicate statistically significant differences according to Tukey’s multiple comparisons test.

To examine the effects of glucose on GPD1 and GPD2 expression, cells were cultured in media containing 0, 1, 5, or 10 mM glucose for 48 h, and protein expression was analyzed by Western blotting. As shown in Figure 3D,E, increasing glucose concentrations in A549 cells induced a dose‐dependent upregulation of GPD2 expression, whereas GPD1 was not detectable. In contrast, in Huh7 cells, increasing glucose levels dose‐dependently upregulated GPD1 protein expression while concomitantly downregulating GPD2. In HepG2 cells, GPD2 expression was reduced under high glucose conditions, whereas GPD1 expression remained largely unchanged. Collectively, these data demonstrate that GPD1 and GPD2 are differentially expressed across A549, Huh7, and HepG2 cells and that glucose availability modulates GPD2 expression in a cell‐type‐specific manner.

### 3.5. GPD2 Inhibition Reduces Proliferation and Migration of HCC Cells

To compare the aggressiveness between HCC lines, the proliferative and migratory capacities of Huh7 and HepG2 cells were examined. HepG2 cells exhibited significantly higher proliferation rates than Huh7 cells over time (Figure S2A). Conversely, wound‐healing assays demonstrated greater migratory capacity in Huh7 cells, with increased wound closure observed at 12 and 24 h compared with HepG2 cells (Figure S2B). These results indicate distinct aggressive phenotypes between the two HCC cell lines, with HepG2 cells displaying enhanced proliferative capacity and Huh7 cells exhibiting greater migratory potential. Huh7 and HepG2 were used for determining the roles of GPD2 in HCC cells.

To determine the role of GPD2 in HCC progression, GPD2 activity was inhibited using KM04416, a potent GPD2 inhibitor, and the effects on HCC cell viability, proliferation, and migration were evaluated. As shown in Figure S3A, KM04416 reduced cell viability in both Huh7 and HepG2 cells in a dose‐ and time‐dependent manner. After 24 h of treatment, the half‐maximal inhibitory concentration (IC_50_) of KM04416 was 42.16 μM in Huh7 cells and 18.59 μM in HepG2 cells. Extending the treatment to 48 h further decreased the IC_50_ values to 13.63 and 9.17 μM in Huh7 and HepG2 cells, respectively, indicating that HepG2 cells were more sensitive to GPD2 inhibition.

The effect of KM04416 on long‐term proliferative capacity was subsequently evaluated over a 5‐day period (Figure S3B). Treatment with 5 and 10 μM KM04416 significantly suppressed the proliferation of Huh7 cells. In HepG2 cells, the inhibitory effect was more pronounced, with 1, 5, and 10 μM KM04416 restricting cell growth to approximately 5‐fold, 2.5‐fold, and 2‐fold, respectively, by Day 5. The effect of KM04416 on HCC cell migration was also examined over 24 h (Figure S3C). Treatment with 10 μM KM04416 significantly reduced the migratory capacity of both Huh7 and HepG2 cells compared with untreated controls. Collectively, these findings demonstrate that pharmacological inhibition of GPD2 with KM04416 suppresses HCC cell viability, proliferation, and migration, supporting a critical role for GPD2 in maintaining the malignant phenotype of HCC.

### 3.6. Metformin Diminishes Aggressive Phenotypes of HCC Cells

Given that metformin could also inhibit GPD2 activity (Figure 2A), we subsequently examined its effects on HCC cell proliferation and migration. The IC_50_ values of metformin for Huh7 and HepG2 cells were first determined using an MTT assay following treatment with metformin for 24, 48, and 72 h. Metformin reduced cell viability in a time‐ and dose‐dependent manner in both cell lines. Notably, Huh7 cells exhibited consistently higher IC_50_ values than HepG2 cells at all time points, indicating that Huh7 cells were less sensitive to metformin treatment (Figure 4A,B). The long‐term effects of metformin on clonogenic survival were further assessed in HepG2 cells using colony formation assays. HepG2 cells were treated with 2, 5, and 10 mM metformin and incubated for 14 days. As shown in Figure 4C, metformin reduced the number of colonies of HepG2 cells in a dose‐dependent manner. To evaluate the impact of metformin on cell motility, wound healing assay was performed in Huh7 and HepG2 cells treated with 2 mM metformin. Metformin significantly impaired migratory capacity in both cell lines, as evidenced by reduced wound closure at 12 and 24 h compared with untreated controls (Figure 4D). Collectively, these results demonstrate that metformin inhibits HCC cell proliferation, clonogenic survival, and migration, supporting its potential role in suppressing aggressive cellular behaviors in liver cancer cells.

**FIGURE 4:**
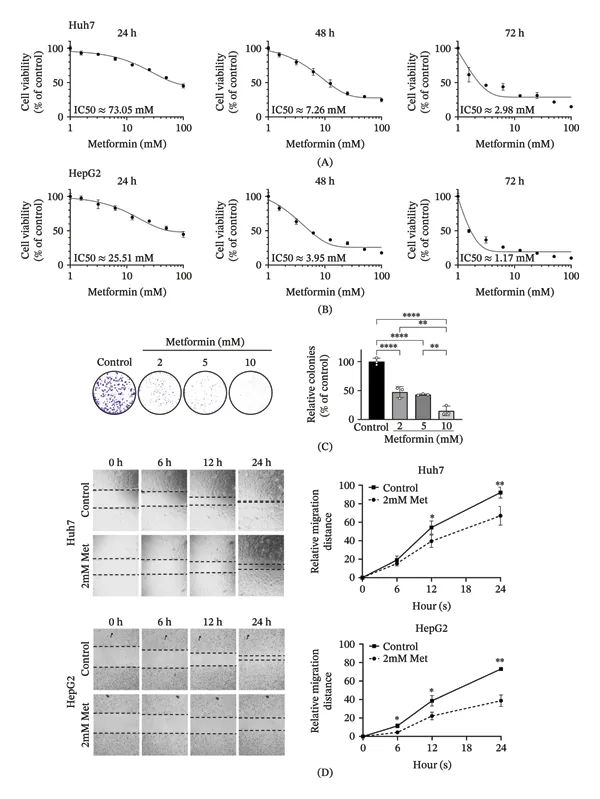
Metformin suppresses the proliferation, clonogenicity, and migration of HCC cells. Dose–response curves showing the effects of metformin on cell viability in (A) Huh7 and (B) HepG2 cells following 24, 48, and 72 h of treatment, as determined by the MTT assay. The half‐maximal inhibitory concentration (IC_50_) values for each treatment duration are indicated in the corresponding graphs. (C) Representative images and quantitative analysis of colony formation in HCC cells treated with metformin (2, 5, and 10 mM). Data are presented as mean ± SEM from three independent experiments; one‐way ANOVA followed by Tukey’s multiple comparisons test (^∗∗^ *p* < 0.01, ^∗∗∗∗^ *p* < 0.0001). (D) Representative wound‐healing images of Huh7 and HepG2 cells treated with 2 mM metformin at the indicated time points (0, 6, 12, and 24 h), with corresponding quantification of relative migration distance. Data are presented as mean ± SEM from three independent experiments. Statistical significance was determined using Student’s *t*‐test (^∗^ *p* < 0.05, ^∗∗^ *p* < 0.01 vs. untreated control).

### 3.7. Inhibition of GPD2 Activity by Metformin or KM04416 Reduces Intracellular ATP Levels in HCC Cells

To investigate whether GPD2 inhibition affects cellular energy metabolism, intracellular ATP levels were measured in Huh7 and HepG2 cells using ATP Assay Kit. As shown in Figure 5A, treatment with metformin markedly reduced intracellular ATP levels in both cell lines, with a decrease of approximately 30% in Huh7 cells and 50% in HepG2 cells relative to untreated controls. Similarly, targeted inhibition of GPD2 with the potent inhibitor KM04416 (GPD2i) significantly diminished ATP production in both Huh7 and HepG2 cells, with reductions exceeding those observed under metformin treatment. However, combining KM04416 with metformin only slightly further reduced ATP production compared with either KM04416 or metformin alone. To further evaluate the effect of the combination on HCC cell viability, an MTT assay was performed after 48 h of treatment. Consistent with the ATP results, the KM04416–metformin combination only marginally decreased Huh7 cell survival and had no additional effect on HepG2 cells. These findings demonstrate that inhibition of GPD2 activity disrupts ATP production in HCC cells and that metformin and KM04416 exert overlapping effects on cellular energy metabolism. The limited additive effect of the combination treatment suggests that both agents may suppress ATP production, at least in part, through a shared GPD2‐dependent mechanism, thereby contributing to the inhibition of HCC cell growth and survival.

**FIGURE 5:**
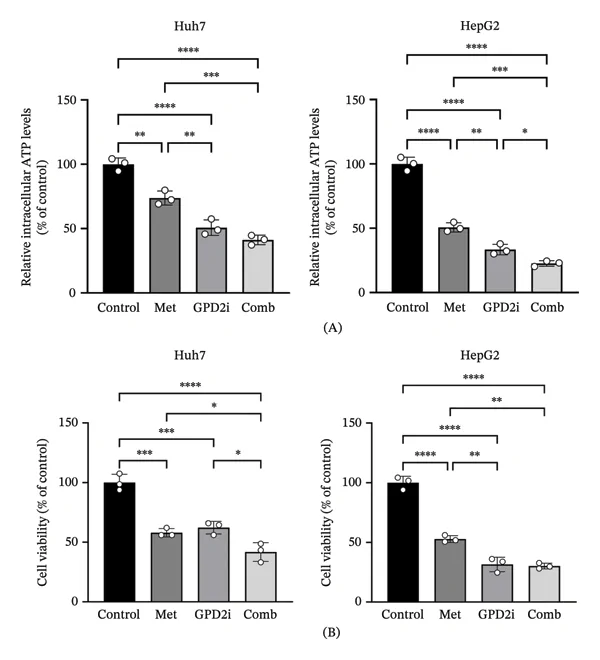
Metformin and KM04416 treatment reduce intracellular ATP in HCC cells. (A) Intracellular ATP levels in Huh7 and HepG2 cells treated with 2 mM metformin (Met) or 10 μM selective GPD2 inhibitor KM04416 (GPD2i) and the combination were determined using an ATP assay kit. (B) Cell viability was determined using MTT assay. Bar graphs show mean ± SEM; ^∗^ *p* < 0.05, ^∗∗^ *p* < 0.01, ^∗∗∗^ *p* < 0.001, and ^∗∗∗∗^ *p* < 0.0001, one‐way ANOVA followed by Tukey’s multiple comparisons test.

### 3.8. Metformin in Low Glucose Conditions Could Better Inhibit HCC Cell Growth

To investigate the effects of metformin on HCC cell proliferation under varying glucose conditions, Huh7 and HepG2 cells were cultured with different glucose concentrations (2, 5, and 20 mM) and treated with increasing doses of metformin (1, 2.5, or 5 mM). As shown in Figure 6A, Huh7 cells treated with 1 mM metformin exhibited reduced proliferation under low glucose (2 mM), with only a modest increase in proliferation over 5 days, while higher glucose concentrations (5 and 20 mM) supported greater proliferation. This glucose‐dependent trend persisted at higher metformin concentrations (2.5 and 5 mM), with increasing suppression of proliferation, particularly under low glucose conditions. At 5 mM metformin, Huh7 proliferation was nearly abrogated at 2 mM glucose. Similarly, in Figure 6B, HepG2 cells demonstrated reduced proliferation in response to metformin treatment, and this effect was exacerbated under low glucose conditions. At 5 mM metformin, cell proliferation was minimal across all glucose concentrations, with the most pronounced suppression at 2 mM glucose. Compared to Huh7, HepG2 cells were generally more sensitive to metformin at lower doses.

**FIGURE 6:**
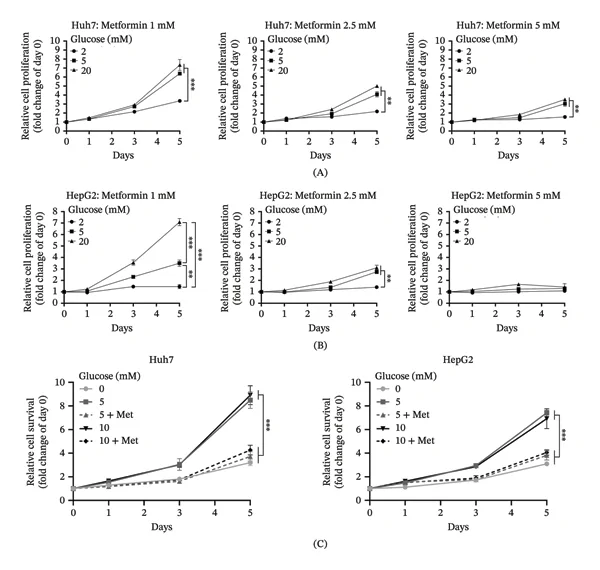
Glucose availability modulates the antiproliferative effects of metformin in hepatocellular carcinoma cells. (A) Huh7 cells were treated with metformin (1, 2.5, or 5 mM) and cultured in media containing 2, 5, or 20 mM glucose. Cell proliferation was monitored for 5 days using the MTT assay and expressed as the fold change relative to day 0. (B) HepG2 cells were treated under the same conditions as in (A), and relative cell proliferation was determined over 5 days. Data are presented as mean ± SEM. Statistical significance was determined using one‐way ANOVA followed by Tukey’s multiple comparisons test. (^∗^ *p* < 0.05, ^∗∗^ *p* < 0.01, ^∗∗∗^ *p* < 0.001). (C) Huh7 and HepG2 cells were cultured in media containing 0, 5, or 10 mM glucose in the presence or absence of 2 mM metformin. Relative cell survival was assessed for up to 5 days using the MTT assay and expressed as the fold change relative to day 0. Data are presented as mean ± SEM from three independent experiments. Statistical significance was determined using two‐way ANOVA followed by Tukey’s HSD test, ^∗∗∗^ *p* < 0.001.

To further assess the interaction between glucose availability and metformin on cell survival, Huh7 and HepG2 cells were cultured in 0, 5, or 10 mM glucose, with or without 2 mM metformin, and cell survival was monitored over 5 days (Figure 6C). In both cell lines, glucose supplementation enhanced cell survival in a dose‐dependent manner. However, metformin significantly inhibited this effect. In Huh7 cells, the addition of metformin under 5 or 10 mM glucose conditions led to a marked reduction in cell viability compared to glucose‐only controls. A similar inhibitory trend was observed in HepG2 cells, although the suppressive effect of metformin appeared slightly less pronounced than in Huh7 cells. These findings indicate that metformin suppresses HCC cell proliferation, with a more pronounced effect observed under glucose‐limited conditions.

### 3.9. Metformin Reduces Prosurvival Signaling, Induces Apoptosis, and Diminishes EMT Progression in a Glucose‐Dependent Manner in HCC Cells

To determine how glucose availability influences the molecular effects of metformin, protein expression levels of cell cycle regulators, apoptotic markers, and epithelial‐mesenchymal transition (EMT)‐related proteins were assessed by Western blotting under normal (5 mM) and high (10 mM) glucose with or without 2 mM metformin. As shown in Figure 7A, high glucose elevated Cyclin D1 expression, while metformin treatment attenuated Cyclin D1 expression in both Huh7 and HepG2 cells, with a more pronounced reduction under low‐glucose conditions. Similarly, the expression of the antiapoptotic protein XIAP was modestly decreased upon metformin treatment, particularly under normal glucose conditions. Markers of apoptosis were notably increased in response to metformin (Figure 7A). Cleaved caspase‐3 levels were elevated upon metformin treatment, especially under normal glucose, indicating enhanced apoptosis under energy stress. In addition, the expression of Slug, a transcription factor involved in the EMT process, increased under high glucose conditions and was suppressed by metformin treatment in both cell lines (Figure 7A), suggesting a potential reversal of mesenchymal traits.

**FIGURE 7:**
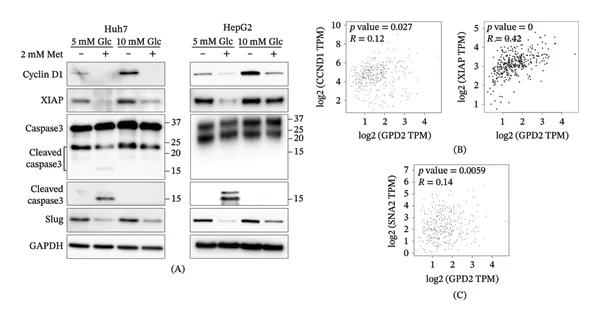
Metformin regulates cell cycle, apoptosis, and EMT‐related proteins in a glucose‐dependent manner. (A) Representative Western blot analysis of Cyclin D1, XIAP, total caspase‐3, cleaved caspase‐3, and Slug in Huh7 and HepG2 cells cultured in media containing 5 or 10 mM glucose (Glc) in the presence or absence of 2 mM metformin (Met). GAPDH was used as the loading control. (B) Pearson correlation analyses between GPD2 and CCND1 (left) or XIAP (right) mRNA expression in hepatocellular carcinoma samples from the TCGA dataset. (C) Pearson correlation analysis between GPD2 and SNAI2 mRNA expression in the TCGA cohort. Correlation coefficients (R) and corresponding *p* values are shown in each plot.

To assess whether the metabolic regulation observed in vitro correlates with clinical cancer transcriptomics data, correlation analyses were performed between GPD2 and metformin‐responsive genes using patient RNA‐seq data via GEPIA. As shown in Figure 7B, GPD2 expression positively correlated with CCND1 and XIAP expression (*R* = 0.12, *p* = 0.027; *R* = 0.42, *p* < 0.001, respectively), suggesting a potential link between mitochondrial metabolism and cell cycle progression. Similarly, Figure 7C shows a positive correlation between GPD2 and SNAI2 expression (*R* = 0.14, *p* = 0.0059), supporting a weak but statistically significant association between GPD2 expression and EMT‐related signaling in HCC tumors. Together, these findings suggest that metformin impairs prosurvival and EMT‐related signaling pathways in a glucose‐dependent manner.

### 3.10. Suppressed Slug Expression Inhibits Migration Ability of HCC Cells

To evaluate the functional role of Slug in HCC cells, Slug expression in Huh7 and HepG2 cells was suppressed using specific siRNA. As shown in Figure 8A, over 80% of Slug protein was suppressed by si‐Slug in both Huh7 and HepG2 cells compared with the scramble control (sc). Cell proliferation was assessed over a 5‐day period (Figure 8B). In Huh7 cells, Slug knockdown significantly suppressed cell proliferation compared with control cells, with a marked difference observed at day 5. In contrast, HepG2 cells showed no significant difference in proliferation between si‐Slug and control groups, indicating a cell line–dependent effect of Slug on cell growth. The effect of Slug silencing on cell migratory capacity was next examined using Transwell migration assays (Figure 8C). Slug knockdown reduced migration to approximately 50% of control in Huh7 cells and 40% in HepG2 cells, indicating that Slug plays a critical role in promoting HCC cell migration. Collectively, these results demonstrate that siRNA‐mediated silencing of Slug reveals its cell‐line‐dependent role in proliferation and its consistent requirement for the migratory ability of both Huh7 and HepG2 cells.

**FIGURE 8:**
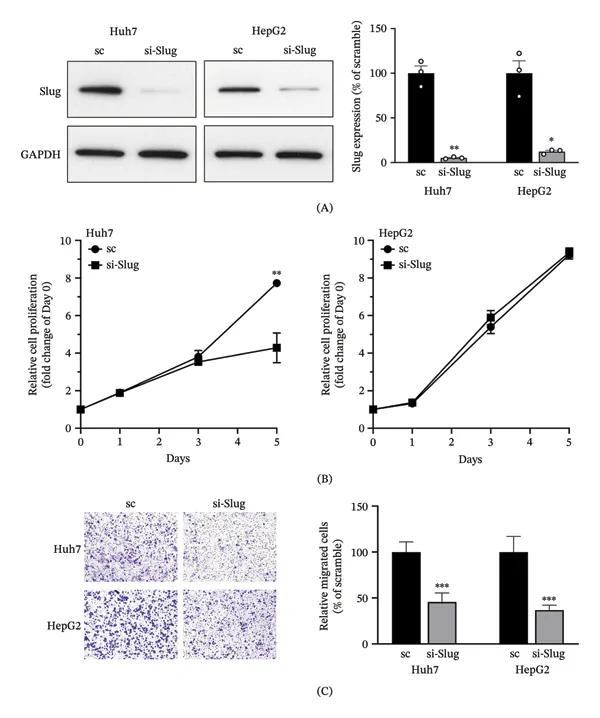
Slug knockdown suppresses the proliferation and migration of HCC cells. (A) Representative Western blot analysis of Slug protein expression in Huh7 and HepG2 cells transfected with scramble control (sc) or Slug‐specific siRNA (si‐Slug). GAPDH served as the loading control. Bar graph presents densitometric quantification of Slug protein expression normalized to GAPDH and expressed as a percentage of the scramble control. (B) Cell proliferation of Huh7 and HepG2 cells transfected with sc or si‐Slug was assessed using the MTT assay over 5 days. (C) Representative images of Transwell migration assays in Huh7 and HepG2 cells transfected with sc or si‐Slug. Bar graph shows the quantification of migrated cells expressed as mean ± SEM from three independent experiments. Statistical significance was determined using Student’s *t*‐test. ^∗^ *p* < 0.05, ^∗∗^ *p* < 0.01, and ^∗∗∗^ *p* < 0.001 vs. the scramble control.

## 4. Discussion

Metabolic reprogramming is a defining feature of hepatocellular carcinoma (HCC), enabling tumor cells to adapt to fluctuating nutrient availability within the liver microenvironment. In this study, we demonstrate that HCC cells exhibit pronounced metabolic plasticity in response to glucose availability and identify mitochondrial GPD2 as a key mediator of this adaptation. Our findings reveal that GPD2 is markedly upregulated in HCC, correlates with poor patient prognosis, and functionally supports glucose‐driven tumor growth and aggressiveness. In addition, the antidiabetic drug, metformin, effectively disrupts GPD2‐linked metabolic, prosurvival, and EMT pathways, particularly under glucose‐limiting conditions.

High glucose significantly enhanced the proliferation and migration of HCC cells, consistent with previous reports linking hyperglycemia to increased cancer aggressiveness and poor prognosis in cancer patients [20–23]. Elevated glucose has been shown to stimulate oncogenic signaling, cell cycle progression, and EMT through PI3K–AKT, MAPK, and HIF‐1α pathways [24–27]. Unlike lung cancer cells, HCC cells retained substantial proliferative capacity under glucose deprivation and were relatively resistant to GLUT1 inhibition. Previous studies have shown that lung adenocarcinoma A549 cells are highly glucose‐sensitive, with glucose deprivation inducing pronounced cellular stress and apoptosis, likely due to insufficient compensation by glutamine‐dependent reductive carboxylation [21, 28–30]. Conversely, HCC cells, particularly Huh7, exhibit strong adaptability to nutrient deprivation by activating alternative metabolic pathways, such as autophagy, lipid metabolism, and the glycerol phosphate shuttle mediated by GPD2 [3, 31, 32]. While A549 cells rely heavily on glucose to sustain rapid proliferation, they display limited metabolic flexibility compared with liver‐derived cancer cells. Together, metabolic plasticity is not a universal feature of all cancers but represents a unique adaptive characteristic of HCC, with GPD2 serving as a critical bypass to maintain energy homeostasis during glucose deprivation.

This study highlights the GPD2, as a key mediator of this metabolic adaptation. In normal liver physiology, GPD2 is essential for glycerol‐derived gluconeogenesis and energy homeostasis [11, 12]. Although dysregulated glycerol metabolism has been implicated in cancer metabolism, its role in HCC progression remains poorly understood. By integrating TCGA, CPTAC, and HPA datasets with functional assays, we demonstrate that GPD2—but not its cytosolic counterpart GPD1—was markedly upregulated at both transcript and protein levels in HCC tissues. In addition, survival analyses based on the TCGA cohort demonstrated that elevated GPD2 expression was independently associated with poor overall survival after adjustment for available clinicopathological variables. These findings support the potential utility of GPD2 as an independent prognostic biomarker in HCC. These data align with emerging evidence that mitochondrial redox shuttles support tumor growth, metabolic flexibility, and metastatic potential [4, 33]. Interestingly, GPD1 expression was reduced in tumors and negatively correlated with GPD2 expression, suggesting functional divergence between the two enzymes during malignant transformation. This observation is consistent with reports indicating tumor‐suppressive roles for cytosolic glycerol metabolism and oncogenic roles for mitochondrial metabolic rewiring [9]. The differential expression of GPD1 and GPD2 between HepG2 and Huh7 cells further supports the concept that distinct HCC subtypes adopt alternative metabolic strategies to balance proliferation and migration, contributing to intratumoral heterogeneity.

Metformin, a widely prescribed antidiabetic drug, has attracted considerable attention for its anticancer effects [13, 34–36]. Metformin has been shown to inhibit tumor initiation and progression in a broad range of malignancies, including bile duct, breast, colorectal, pancreatic, lung, and prostate cancers, through both systemic and cell‐autonomous mechanisms [13, 37, 38]. Epidemiological studies have demonstrated a reduced incidence of HCC and improved survival outcomes among diabetic patients treated with metformin [39, 40]. Notably, recent studies have identified GPD2 as a mitochondrial target of metformin, linking its anticancer activity to the inhibition of the glycerol‐3‐phosphate shuttle and redox imbalance [14, 15]. Our results extend these findings by demonstrating that metformin suppresses HCC cell proliferation, clonogenic survival, and migration in a glucose‐dependent manner, with enhanced efficacy under low‐glucose conditions. These observations suggest that metformin is particularly effective when HCC cells rely on mitochondrial metabolic plasticity under limited glucose availability.

At the molecular level, metformin reduced Cyclin D1 and XIAP expression while increasing caspase‐3 cleavage, indicating inhibition of cell cycle progression and activation of apoptosis. Similar effects of metformin on cell cycle arrest and apoptotic signaling have been reported in several cancers [41–43]. Importantly, these effects were more pronounced under low‐glucose conditions, supporting the concept that energetic stress sensitizes cancer cells to mitochondrial inhibition. Metformin also reduced the expression of Slug, a key EMT transcription factor associated with HCC migration and invasion. EMT suppression by metformin has been linked to AMPK activation and inhibition of TGF‐β, Wnt/β‐catenin, STAT3, and NF‐ĸB signaling pathways [36, 44, 45]. Consistently, our correlation analyses revealed positive associations between GPD2 expression and CCND1, XIAP, or SNAI2 expression in human HCC tumors, supporting potential associations between mitochondrial metabolism, cell cycle regulation, and EMT‐related signaling. Notably, although the association between GPD2 and SNAI2 reached statistical significance, the relatively weak correlation suggests a correlative association rather than evidence of a direct mechanistic link between GPD2 and EMT signaling. In addition, the downregulation of Slug may contribute to reduced migratory capacity of HCC cells while selectively inhibiting HCC cell growth.

This study provides novel evidence positioning GPD2 as both a prognostic biomarker and a metabolic vulnerability in HCC; however, several limitations should be acknowledged, as our findings primarily rely on in vitro models and integrative bioinformatic analyses. Future research should bridge these gaps through advanced biological modeling—including in vivo validation using xenograft or genetically engineered mouse models—to confirm GPD2’s role in tumor progression and therapeutic response, with genetic manipulation establishing direct causal links to metabolic adaptation and glucose deprivation bypass in HCC. Importantly, the current evidence linking metformin responses to GPD2 is based primarily on pharmacological approaches. Although KM04416 phenocopied several effects of metformin and the combination of KM04416 and metformin produced limited additional effects on ATP levels and cell viability, these findings do not establish a direct or exclusive GPD2‐dependent mechanism. Potential off‐target effects of KM04416 and convergence on downstream energetic endpoints cannot be excluded. Therefore, genetic validation, including GPD2 knockdown with rescue experiments and/or GPD2 overexpression to assess whether GPD2 levels modulate metformin sensitivity, will be required to establish a direct causal role for GPD2 in mediating the effects of metformin. Although two HCC cell lines with distinct biological characteristics (HepG2 and Huh7) yielded consistent findings, the use of A549 lung adenocarcinoma cells as a comparator does not establish that the observed glucose‐deprivation tolerance is specific to HCC relative to nonmalignant hepatocytes. Further validation using additional HCC cell lines and nonmalignant hepatocyte models, such as LO2 cells or primary human hepatocytes, will be needed to establish the generalizability and HCC specificity of these findings. Beyond the lab, investigating GPD2 expression as a predictive biomarker for sensitivity to metformin or other mitochondrial‐targeting agents is essential, particularly to assess correlations with clinical outcomes and explore metabolic differences across HCC subtypes (as suggested by divergent GPD1/GPD2 patterns in HepG2 vs. Huh7 cells) for refined, personalized treatment strategies. The concentrations of metformin used in this study are higher than clinically achievable plasma concentrations. Moreover, tissue accumulation of metformin in vivo is not fully recapitulated under conventional cell culture conditions. Therefore, caution should be exercised when extrapolating these findings to the clinical setting, and future studies using physiologically relevant models and in vivo systems are warranted to validate the role of GPD2 in mediating the antitumor effects of metformin.

## 5. Conclusions

In summary, our findings suggest that GPD2‐mediated glycerol metabolism may contribute to the metabolic adaptation of HCC cells under glucose‐limited conditions and identify metformin as a potential inhibitor of this pathway. These findings support a potential role for mitochondrial metabolic plasticity in HCC adaptation and warrant further investigation to determine whether GPD2 may represent a therapeutic target for HCC.

## Author Contributions

Supakant Siripongsampan, Manusanun Kateyai, Kanadit Piriyapairoje, and Jantanipa Hannam: methodology, formal analysis and investigation, and writing–review and editing; Charupong Saengboonmee: methodology, writing–review and editing, and resources; Teeranai Ittiudomrak: methodology and writing–review and editing; Sukanya Jaroenporn: conceptualization, methodology, writing–review and editing, and resources; Sittiporn Pattaradilokrat: conceptualization, methodology, writing–review and editing, and resources; Chatchai Phoomak: conceptualization, methodology, writing–original draft preparation, funding acquisition, and supervision.

## Funding

This work was funded by the Thailand Science Research and Innovation Fund, Chulalongkorn University (HEA_FF_68_200_2300_046) to Chatchai Phoomak.

## Conflicts of Interest

The authors declare no conflicts of interest.

## Supporting Information

Additional supporting information can be found online in the Supporting Information section.

## Supporting information


**Supporting Information** Supporting information includes the following figures and table: Supporting Figure S1: dysregulation of glycerol‐derived gluconeogenesis and glycogen metabolism genes across cancers and their association with patient survival. Supporting Figure S2: distinct proliferative and migratory phenotypes of Huh7 and HepG2 cells. Supporting Figure S3: effects of GPD2 inhibition on proliferation and migration in HCC cells. Table S1: list of antibodies used for Western blot analysis and their corresponding experimental conditions.

## Data Availability

All data needed to evaluate the conclusions in the paper are present in the paper and/or the Supporting Information.
