# Supplementary material for: Metformin Attenuates Glucose‐Driven Hepatocellular Carcinoma Progression by Targeting GPD2‐Mediated Metabolic Plasticity
Source: Scientifica (Cairo). 2026 Oct 27;2026:9372425. doi: 10.1155/sci5/9372425 (PMC13711023; doi:10.1155/sci5/9372425)
Supplement: Supplementary file 1 [file SCI5-2026-9372425-s001.pdf]

SUPPLEMENTARY DATA

Metformin Attenuates Glucose-Driven Hepatocellular Carcinoma Progression by Targeting GPD2-Mediated Metabolic Plasticity

Supakant Siripongsampan[1a], Manusanun Kateyai[1a], Kanadit Piriyapairoje[1], Jantanipa Hannam[2], Charupong Saengboonmee[3], Teeranai Ittiudomrak[1,4], Sukanya Jaroenporn[1], Sittiporn Pattaradilokrat[1], Chatchai Phoomak[1,4]*

[1] Department of Biology, Faculty of Science, Chulalongkorn University, Bangkok 10330 Thailand

[2] Bioinformatics and Computational Biology Program, Graduate School, Chulalongkorn University, Bangkok 10330 Thailand

[3] Department of Biochemistry, Faculty of Medicine, Khon Kaen University, Khon Kaen 40002 Thailand

[4] Center of Excellence in Systems Biology, Faculty of Medicine, Chulalongkorn University, Bangkok 10330 Thailand

[a] These authors contributed equally to this work.

*** Corresponding author:**

Chatchai Phoomak, Ph.D

Department of Biology, Faculty of Science, Chulalongkorn University,

254 Phaya Thai Rd, Pathum Wan, Bangkok 10330 Thailand

Email: Chatchai.ph@chula.ac.th

Supplementary Data:

Suppl Fig S1. Dysregulation of glycerol-derived gluconeogenesis and glycogen metabolism genes across cancers and their association with patient survival.

Suppl Fig S2. Distinct proliferative and migratory phenotypes of Huh7 and HepG2 cells.

Suppl Fig S3. Effects of GPD2 inhibition on proliferation and migration in HCC cells.

Table S1. List of antibodies used for Western blot analysis and their corresponding experimental conditions.

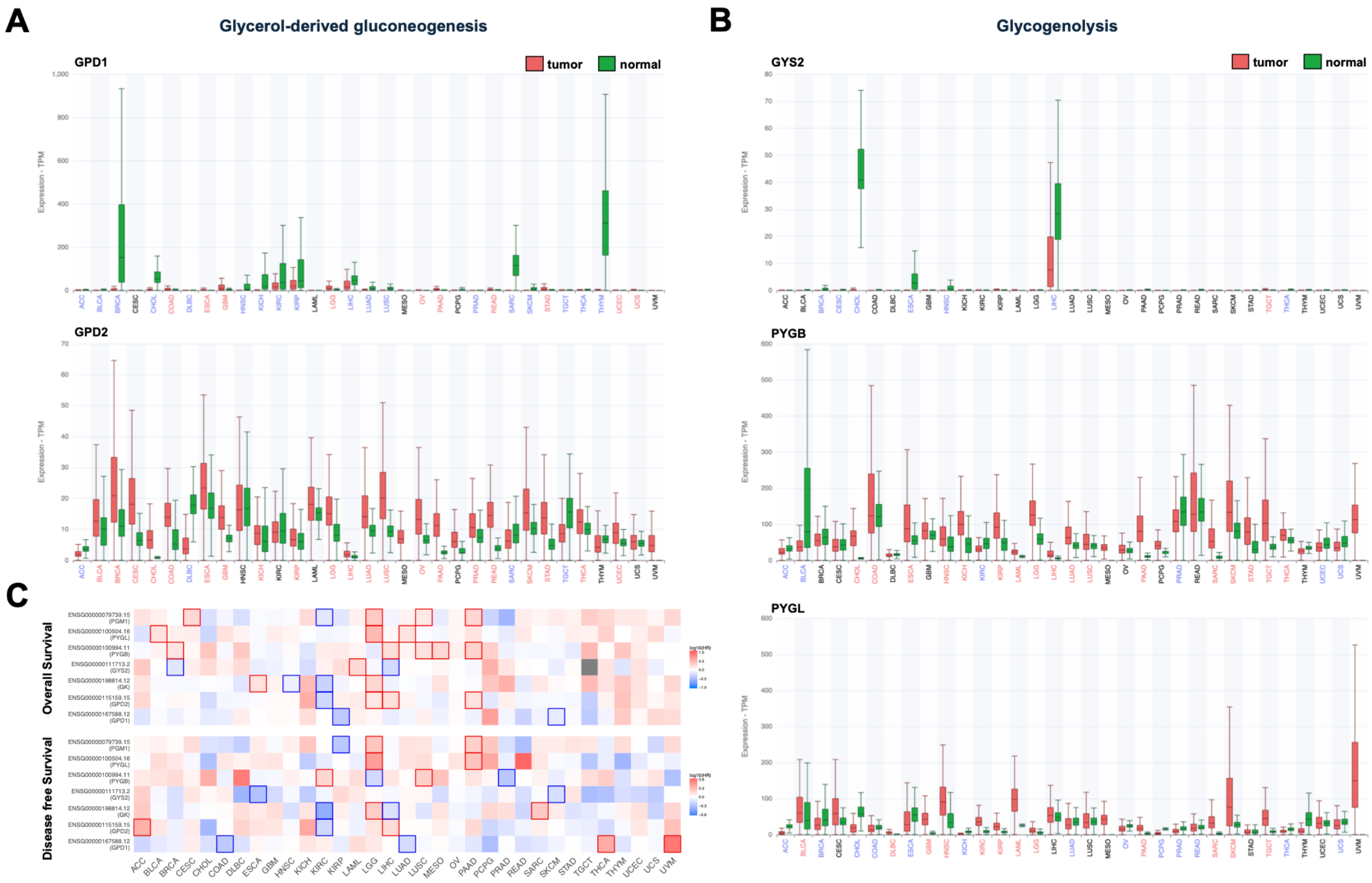

Supplementary Figure S1. Dysregulation of glycerol-derived gluconeogenesis and glycogen metabolism genes across cancers and their association with patient survival. A) GEPIA analysis of GPD1 and GPD2 expression in tumor and normal tissues. B) Expression of glycogen metabolism genes (GYS2, PYGB, PYGL) across cancers. (Blue text = significantly upregulated in normal, Red text = significantly upregulated in tumor) C) Heatmap showing associations between gene expression and overall survival (top) and disease-free survival (bottom) across cancer types. Red and blue indicate positive and negative associations with survival, respectively; boxed-cells denote statistically significant associations. *Footnote:* Cancer types analyzed include adrenocortical carcinoma (ACC), bladder urothelial carcinoma (BLCA), breast invasive carcinoma (BRCA), cervical squamous cell carcinoma and endocervical adenocarcinoma (CESC), cholangiocarcinoma (CHOL), colon adenocarcinoma (COAD), esophageal carcinoma (ESCA), glioblastoma multiforme (GBM), head and neck squamous cell carcinoma (HNSC), kidney chromophobe (KICH), kidney renal clear cell carcinoma (KIRC), kidney renal papillary cell carcinoma (KIRP), liver hepatocellular carcinoma (LIHC), lung adenocarcinoma (LUAD), lung squamous cell carcinoma (LUSC), lymphoid neoplasm diffuse large B-cell lymphoma (DLBC), mesothelioma (MESO), ovarian serous cystadenocarcinoma (OV), pancreatic adenocarcinoma (PAAD), prostate adenocarcinoma (PRAD), rectum adenocarcinoma (READ), sarcoma (SARC), skin cutaneous melanoma (SKCM), stomach adenocarcinoma (STAD), testicular germ cell tumors (TGCT), thyroid carcinoma (THCA), thymoma (THYM), uterine corpus endometrial carcinoma (UCEC), uterine carcinosarcoma (UCS), and uveal melanoma (UVM).

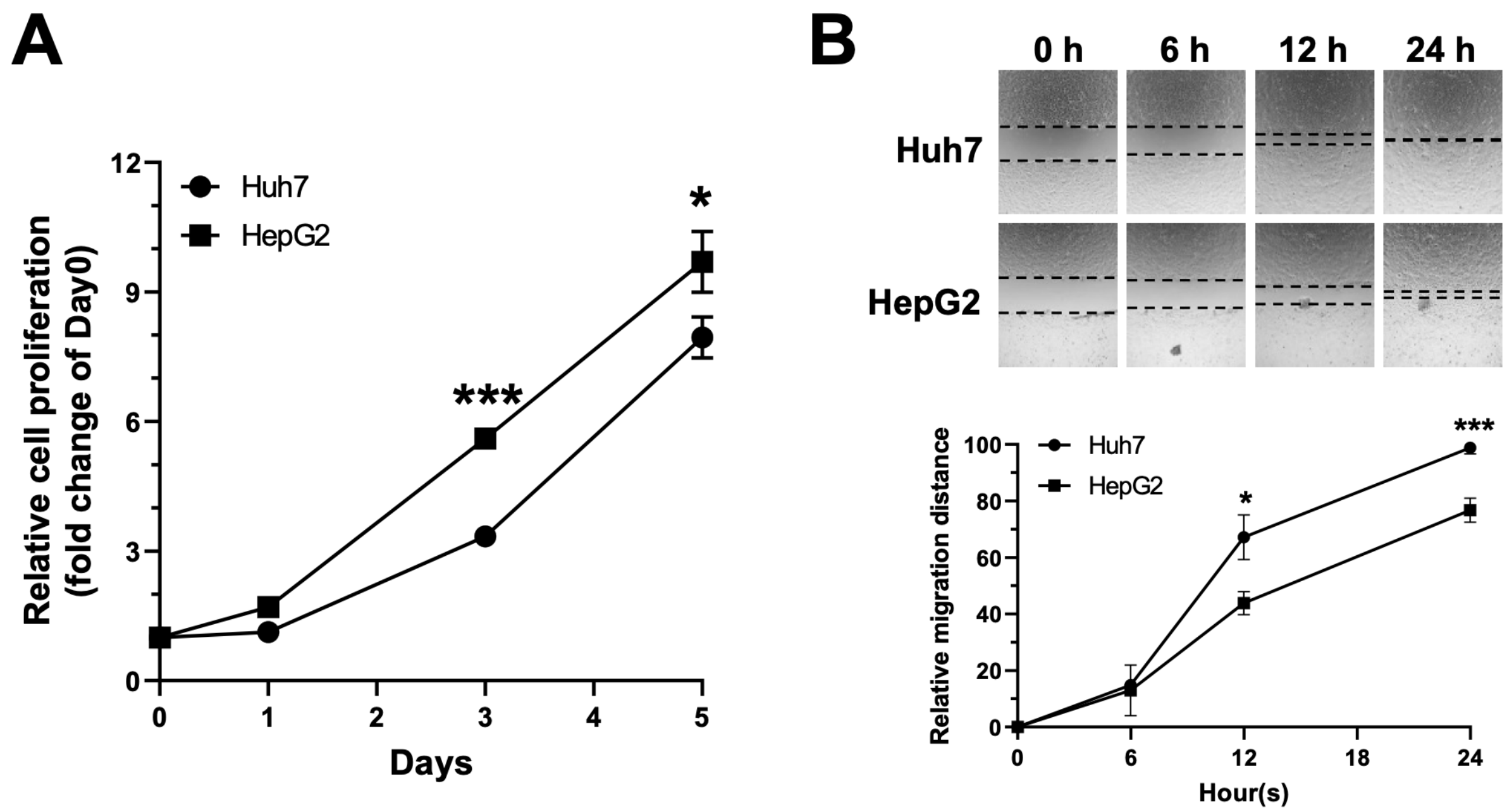

Supplementary Figure S2. Distinct proliferative and migratory phenotypes of Huh7 and HepG2 cells. A) Relative cell proliferation of Huh7 and HepG2 cells over 5 days. B) Wound healing assays assessing cell migration at the indicated time points. Dashed lines indicate wound edges. Data are presented as mean ± SEM from three independent experiments. *$P < 0.05$, ***$P < 0.001$, Student's *t*-test.

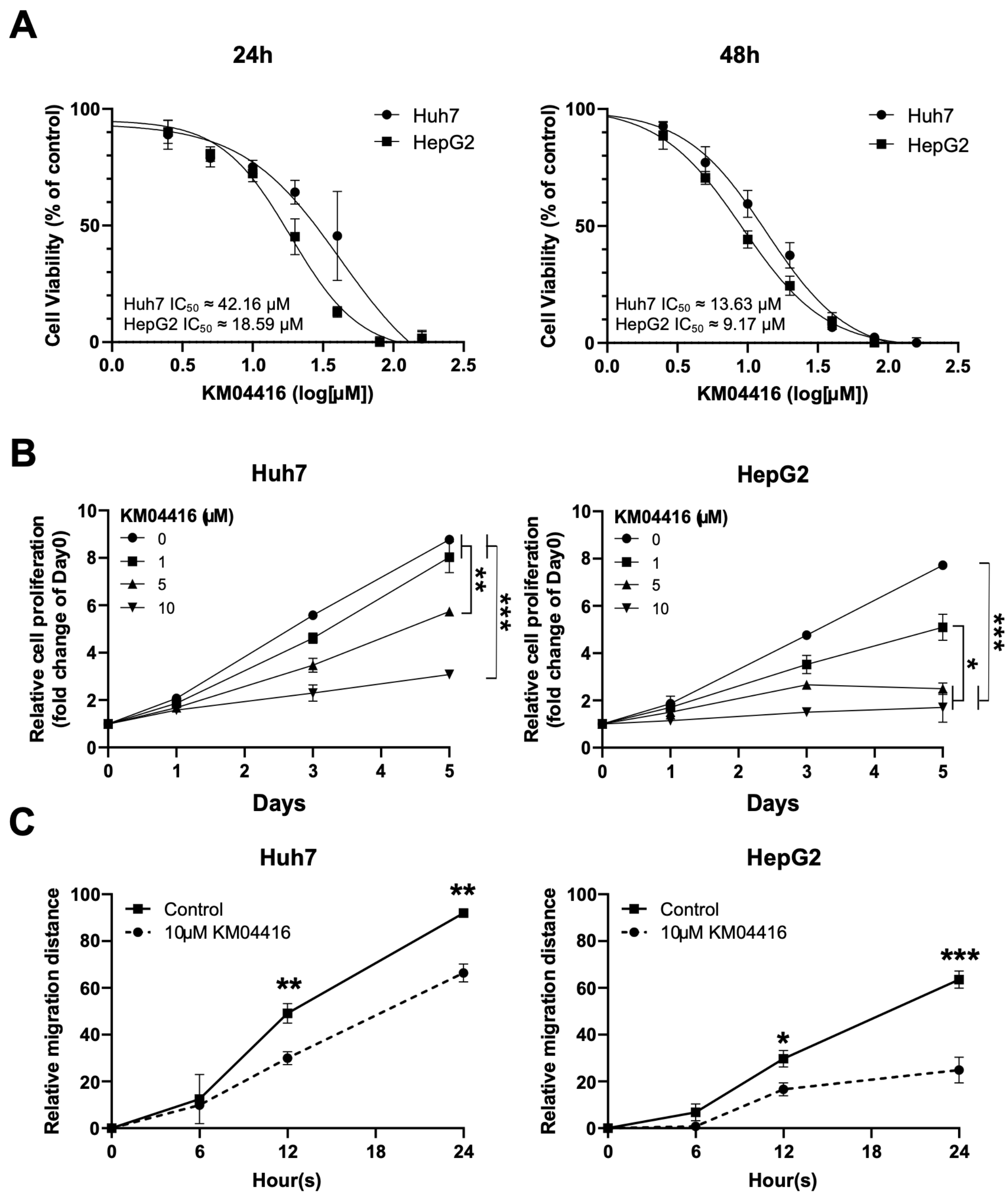

Supplementary Figure S3. Effects of GPD2 inhibition on proliferation and migration in HCC cells. A) Sigmoidal dose-response curves demonstrated dose-dependent inhibition of cell viability by KM04416 (GPD2 inhibitor) in Huh7 and HepG2 cells after 24 and 48 hours of treatment. B) Proliferation of Huh7 and HepG2 cells over 5 days in the presence of 0, 1, 5, or 10 µM KM04416. C) Migration of Huh7 and HepG2 cells over 24 hours following treatment with 10 µM KM04416 compared with untreated controls, expressed as relative migration distance. Data are presented as mean ± SD; * $P < 0.05$, ** $P < 0.01$, *** $P < 0.001$ *vs.* control, Student's *t*-test or ANOVA.

Table S1. List of antibodies used for Western blot analysis and their corresponding experimental conditions.

**Antibody (Clone)**	**Cat No.**	**Company**	**Working dilution**
anti-GPD1	13451-1-AP	Proteintech, Rosemont, IL	1:1,000
anti-GPD2	17219-1-AP	Proteintech, Rosemont, IL	1:1,000
anti-GAPDH	AC002	Abclonal, Düsseldorf, Germany	1:5,000
anti-cyclin D1 (E3P5S)	55506	Cell signaling, Danvers, MA	1:1,000
anti-XIAP (H-202)	sc-11426	Santa Cruz, Dallas, TX	1:500
anti-caspase-3 (D3R6Y)	14220	Cell signaling, Danvers, MA	1:1,000
anti-cleaved caspase-3 (Asp175) (5A1E)	9664	Cell signaling, Danvers, MA	1:500
anti-slug (C19G7)	9585	Cell signaling, Danvers, MA	1:1,000
